# Ubiquitous Virtual Private Network: A Solution for WSN Seamless Integration

**DOI:** 10.3390/s140100779

**Published:** 2014-01-06

**Authors:** David Villa, Francisco Moya, Félix Jesús Villanueva, Óscar Aceña, Juan Carlos López

**Affiliations:** Department of Technology and Information Systems, School of Computer Science, University of Castilla-La Mancha, Altagracia 50, Ciudad Real 13071, Spain; E-Mails: francisco.moya@uclm.es (F.M.); felixjesus.villanueva@uclm.es (F.J.V.); oscar.acena@uclm.es (O.A.); juancarlos.lopez@uclm.es (J.C.L.)

**Keywords:** wireless sensor network, sensor actuator network, virtual network, network-integration

## Abstract

Sensor networks are becoming an essential part of ubiquitous systems and applications. However, there are no well-defined protocols or mechanisms to access the sensor network from the enterprise information system. We consider this issue as a heterogeneous network interconnection problem, and as a result, the same concepts may be applied. Specifically, we propose the use of object-oriented middlewares to provide a virtual private network in which all involved elements (sensor nodes or computer applications) will be able to communicate as if all of them were in a single and uniform network.

## 1. Introduction

**Figure 1.**
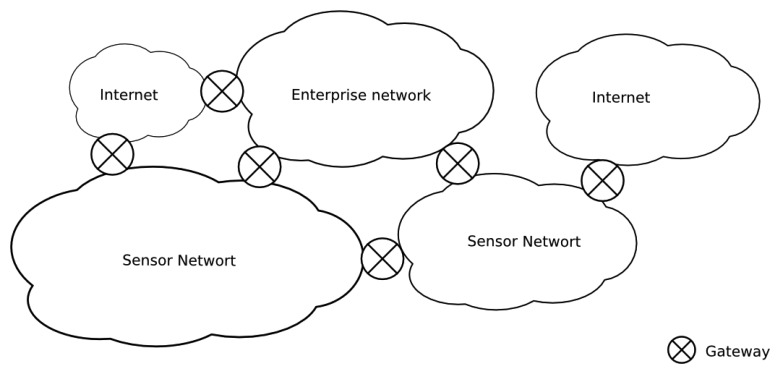
Integrating infrastructure should not constrain the topological structure.

The integration of sensor networks in enterprise information systems is still problematic and unnatural. The interaction between applications and sensor services usually requires specific mechanisms that are often centralized on application-level gateways. Usually, the access to sensor data in the literature is performed through a single node with two interfaces, one for the sensor data (e.g., a 802.15.4 wireless interface) and another for the enterprise information network (e.g., the Ethernet). In many situations, this gateway presents specific software for the adaptation of the protocol stack from the sensor domain to the enterprise domain. However, this type of infrastructure implies a single point of failure. From an engineering point of view, a single gateway implies a single point of failure, associated with a specific application, which could lead to a difficult and inefficient network topology; *i.e.*, the network topology between the domains could be arbitrary and not dependent on the logical integration infrastructure (Figure 1).

At the logical level, this type of infrastructure is inconvenient for the actor/actuator nodes (hereinafter, the term “sensor node” is applied to refer to any actor/actuator devices.) and complicates, or just makes impossible, free interaction among applications and sensor nodes and, particularly, interaction among sensor nodes.

This paper introduces a novel approach to achieve a more flexible and decoupled way to provide and request sensor services that support several gateways among sensor and enterprise domains (sometimes called a *multi-sink*). By means of a common application-level protocol, the sensor nodes and the applications can interact in any scenario, even among sensor nodes belonging to remote networks.

Instead of designing a new application protocol from scratch, we focus our attention on the protocols used by object-oriented middlewares, *i.e.*, CORBA (Common Object Request Broker Architecture) or ZeroC Ice (Internet Communication Engine). These middlewares have traditionally been used in scalable and efficient distributed heterogeneous applications, so we start with well-known and tested protocols. The application protocol used in these middlewares is able to marshall and unmarshall invocation messages between distributed objects. These types of protocols are already integrated into wireless sensor networks [1], but always inside of the same sensor network domain. Of course, this makes it possible to use an object-oriented middleware, which transforms the sensor network integration in the case of distributed heterogeneous programming.

## 2. Related Work

**Figure 2.**
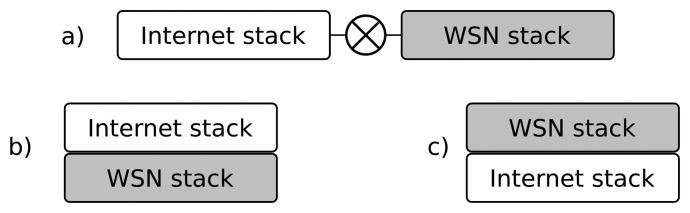
Architectures for integrating sensor networks with the Internet: (**a**) application gateway or NAT (Network Address Translation); (**b**) IP overlaying sensor network and (**c**) sensor network overlaying IP.

In a way, H. Dai [2] aims at similar goals: “Unlike application-level gateways, that require semantic knowledge of each application in order to make a routing decision, the overlay gateway routes based on sensor network layer information”. They propose an *overlay network* to interconnect applications with sensor nodes, extending the sensor network internal protocol over the Internet. This process functions as a *virtual sensor network* thanks to *overlay gateways*. In these researchers' words: “It is a sensor network overlaying IP (Internet Protocol)” (see Figure 2). The gateway encapsulates the sensor network protocol packets (including the network; and the transport and the application headers) on the TCP (Transport Control Protocol) or the UDP (User Datagram Protocol) segments. As the sensor network stack is preserved, the components at hosts (the virtual sensors) must process all of these *strange* headers at the application layer to maintain the illusion of a single flat network.

SenseWrap [3] follows another approach. In it, *virtual sensors* are referred to the wrapped versions of the actual sensors. It focuses on self-configuration providing a standard Zeroconf to discover and find the sensor services. It is a middleware to get *IP overlaying to the sensor network*. SensorWrap is, in many senses, similar to OSGi (Open Services Gateway Initiative) [4], which is the paradigm of a semantic application-level single gateway and, therefore, the model that we try to avoid. Tenet [5] is a more sophisticated network architecture that divides the sensor network into a set of tiers. Each tier has a master and several nodes. Most of the processing and application-specific tasks run in the masters. Hence, it is a multi-gateway approach that avoids a single point of failure, but may significantly degrade the network performance if some of the masters fail. Other approaches, such as [6], provide *families* of middlewares to address heterogeneity, although they are more geared toward solving the problem with devices rather than communications.

The “all over IP” approach could solve the problem, but introduces overhead, even in the most the low-footprint implementations (*i.e.*, uIP (microIP) [7]) and is not affordable for some sensor domains. Other protocols, such as message queue telemetry transport protocol [8] (MQTT) from IBM, are for telemetry applications, so they do not support actuators and individual sensor-to-actuator interactions. 6LowPAN (IPv6 over Low power Wireless Personal Area Networks), CoAP (Constrained Application Protocol) and similar protocols follow the same idea. They imply additional overheading and do not avoid using protocol translation between the sensor network and the trunk network.

As we will see in the following sections, our approach uses the network and the transport protocol stack most appropriate at each domain; we use a common protocol only at the application level. As far as we know, none of the existing approaches provide an integrating mechanism with such flexibility and, integrating in a transparent way, with different domains and applications.

### 2.1. Virtual Networks

Let us focus the problem in a more generic way. Network interconnection (between sensors, trunk networks, *etc.*) is feasible when both use the same protocol, at least at the network layer. However, in practice, there are many scenarios and applications that impose specific/proprietary/non-compatible protocols on the sensor network. There are many causes for this (*i.e.*, energy efficiency, real time characteristics, *etc.*), but we will not analyze them here. Under these circumstances (the usual case), application-level gateways are used. Our concept is quite similar to the conventional *virtual private network*. That is, *hosts* suitable for interchanging information have identifiers in the same address space, creating the illusion that they are all neighbors, although a portion of them are remotely connected through other networks. Note that our work is *not* related with the virtual sensor network (VSN) concept. VSN [9] is a mechanism to select (sometimes dynamically) a subset of sensors and provide them to the user/task as a different (virtual) network, so it is an issue focused on providing sensor node logical aggregation, which is not to be confused with a virtual private sensor network (VPSN). A VPSN [10] is *virtual* in another sense. It provides a per-user sensor network vision. Neither of them are related to network interconnection issues. Perhaps these are not the more suitable names for their claimed purposes.

The conventional VPN protocols in the TCP/IP world work mostly at two levels:

- Link level: The VPN may expose a diffusion link or LAN (Local Area Network), such as a virtually switched Ethernet, in which everyone receives all broadcast frames or even multicast frames, independently of where they are. From the operating system point of view, a virtual NIC (Network Interface Controller) is provided.
- Network level: The alternative is to emulate an IP network (or other network layer protocol). In this case, all the hosts have IP addresses of the same address block, usually private addresses. Any host may send IP packages to any other “neighbor”. The mechanism is usually provided as a *point-to-point* network interface.

In both cases, the involved hosts may have conventional/physical network interfaces. Additionally, in both cases, the responsible device for maintaining the illusion is called the *VPN switch*, but despite its name, it is a pure software engine, although there are many commercial solutions based on physical devices for practical convenience.

### 2.2. Object-Oriented Communication Middlewares

**Figure 3.**
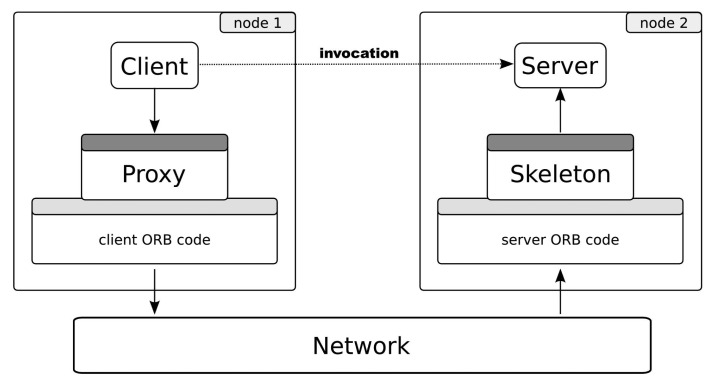
Object-oriented middleware invocation mechanism.

Object-oriented communication middleware has been a well-known concept for more than ten years. Some examples are CORBA [11], ZeroC Ice [12], Java RMI (Remote Method Invocation) [13], *etc.* Figure 3 shows the essential behavior of this type of middleware. From a programmer point of view, the invocation occurs as usual in the object-oriented paradigm (dotted arrow). In reality, it does not happen in this way. The client invokes over a reference of the remote object: the *proxy*. Using the communication core, the invocation is coded (the *marshalling* process) to the specific binary protocol and transmitted to a server using the underlying network. At the server side, the invocation is re-built (*unmarshalling*) and finally arrives at the object; the reply goes back to the client in the same manner.

The server side implements a well-defined *interface* shared by the clients. Interfaces are usually defined using an interface description language, such as IDL (Interface Definition Language) in CORBA or Slice in Ice. That is, the concrete invocation message format depends on a set of interfaces specified by each distributed application designer. Both the proxy and the skeleton parts are dependent on the interface and may be generated by tools, usually provided by middleware vendors.

This communication is feasible thanks to the object request broker (ORB), or just *broker*. The ORB is usually a library that provides to clients transparent references to remote objects (proxies). The interaction between the client and the object requires data communication between local and remote ORBs. To assure interoperability, the protocol and the rules to communicate these ORBs (the inter-ORB protocol) must be standardized.

In any case, the middleware provides a uniform, generic and fully specified application protocol to transport invocation messages and their corresponding replies, errors, *etc.* We can say that the middleware protocol is more similar to an *invocation transport protocol* than an application protocol. We propose the *middleware protocol* as a common message format for all of the entities participating in the communication (sensor node or applications).

### 2.3. picoObjects

The picoObject approach [1] is a previous work that makes it possible to implement conventional distributed objects on network-embedded nodes with a minimal footprint. This is the ability of the sensor network nodes (or similar platforms) to directly process, send and reply standard inter-ORB invocation messages. The present work assumes that all of the sensors or actuators in the nodes are provided as distributed objects by means of picoObjects. This approach was proven to be applicable to several conventional object-oriented middlewares (CORBA, ZeroC Ice and even web services), and it does not imply a loss of generality.

## 3. Ubiquitous Virtual Private Network (UVPN) for Sensor Networks

Our approach, called the ubiquitous virtual private network (UVPN), uses the same VPN concept available in TCP/IP networks, although implemented at a higher abstraction layer. Each *host* may have one or more *object adapters*. An object adapter is responsible for exposing local *objects* to the network. Each object adapter is accessible through *endpoints*, *i.e.*, logical network connection points. In the general purpose middlewares, these endpoints provide support for the TCP/IP protocol suite, and they are associated with IP addresses and ports by means of sockets or other programming artifacts. Emulating the conventional VPN model, we assign homogeneous addresses to all the involved components, regardless of whether they are physical sensor nodes or PC applications. To achieve this, we require a new type of endpoint (a *UVPN endpoint*), encapsulating the specific addressing. Note that the UVPN endpoint, as the conventional VPN counterpart, uses the same underlying transports, *i.e.*, TCP, UDP or SSL, in the Internet case. We are using ZeroC Ice [14] in our current prototypes, although any other object-oriented middleware could be used instead. Sensor nodes with a minimal footprint are able to process application messages using the underlying middleware protocol (IceP (Ice Protocol) for Ice [14]) thanks to the picoObject approach.

Of course, these virtual logic addresses need to be mapped to the corresponding underlying address scheme by some translation mechanism (equivalent to neighbor discovery in conventional protocols). Because this type of translation may be expensive and complex for a sensor network, our approach here uses a shared address scheme (not translated or mapped) to the node link technology. In this way, the endpoint can deliver the invocation directly to the corresponding node. Obviously, this shortcut couples the protocol with a concrete physical communication technology, but even so, it is an interesting improvement to achieve a seamless network interconnection. Our approach may be perceived as a virtual private network, because the components outside the sensor network have addresses belonging to the sensor network space address. Regardless, this limitation may be overcome by defining a general purpose address scheme, assuming the cost of the appointed address mapping. This more ambitious approach is now a work in progress.

Although conventional gateways and UVPN switches are physically similar (both are devices connected to two or more heterogeneous networks), they have very different behaviors. Conventional gateways expose sensors to the trunk network applications as virtual artifacts (delegates) using the protocol and the specific communication technology of the trunk network (for example, web services). A software service at the gateway provides these delegates and addresses the sensor network using a specific protocol (example: Crossbow motes). The gateway is a stateful engine, and this makes the multi-sink complex.

The UVPN switch just forwards the middleware protocol messages (for example, GIOP (General Inter-ORB Protocol) or IceP) between the switch ports using the source and the destination addresses. In some aspects, its behavior is similar to that of an Ethernet bridge switching frames among heterogeneous LANs. With our approach, both applications and devices can address the middleware messages. The UVPN switch is stateless, and this makes it possible to cause several of them to interconnect two networks, providing redundancy. Certainly, this redundancy causes cycle problems, but it can be solved using the same techniques typical of switched networks.

UVPN along with the middleware protocol provides a good level of interoperability among heterogeneous networks. The source node (client) creates a standard middleware request that is encapsulated in the corresponding protocol stack (most likely TCP/IP). The request arrives to the UVPN switch, and it is unencapsulated. The switch determines the destination node (depending on the UVPN address) and encapsulates the request using the destination port stack; the reply message is managed in the same way. This is similar to the behavior of an IP gateway or an Ethernet hybrid bridge. Note that both the source of the request (the client) and destination (the object) can be computer applications or WSN nodes.

### 3.1. Object Proxies

The *object proxy* is a usual concept in object-oriented middlewares. It is a delegate, a local object (in the client memory space) that transparently forwards received invocations to the actual object in a remote location. The proxy encapsulates the details to connect and send messages to the corresponding remote object. These details include the object identity (a globally unique identifier) and a set of endpoints. For example, in the ZeroC Ice middleware, the string representation of a typical proxy may be “OBJ1 -t:tcp -h example.com -p 2020”, where “OBJ1” is the object identity and “example.com:2020” is a remote passive TCP socket.

The UVPN endpoint has its own communication details. An example of a proxy using the UVPN endpoint would be “OBJ2 -d:uvpn -h 0x01”, where “0x01” is the *native* sensor node address. It works on the other side, too; that is, objects in a PC connected to the trunk network are accessible through the same type of address. We call them “virtual nodes”; the virtual node may hold sensors and actuators fully indistinguishable from its actual counterparts. In [2], a *virtual sensor* is defined as “any entity that communicates with peer entities on a real sensor network through a common set of protocols, network layer and above”. We take this definition with the following alteration: it is not required to have the same set of protocols. In UVPN, only the application-layer protocol (the inter-ORB) and the addressing scheme must be shared; the underlying protocols may be absolutely different. Note that the virtual nodes may hold *sensors*/*actuators*, but also may have pure clients, that is, applications that only makes queries or produce events.

### 3.2. Functional Components

The main component of the UVPN architecture is the *UVPN switch*, a service that should reside in a host that has physical interfaces to both networks: sensor and trunk. Its goal is similar to a conventional *VPN switch*. The switch knows all compatible remote object adapters; that is, those that have an endpoint supporting UVPN addressing. For this, each of these adapters must register themselves in the *switch*. The switch is a conventional distributed object implementing the UVPN::Switch interface shown below:
module UVPN { class Address {}; interface Transceiver { void send(Address addr, ByteSeq payload); }; interface Switch extends Transceiver { void add(Address addr, Transceiver* prx); void remove(Address addr); Transceiver* find(Address addr); };};

For better transparency, the UVPN endpoint (on the conventional computer side) performs the registration on behalf of the adapter. When an endpoint is instantiated, it invokes the Switch.add() method in the designated remote switch to bind a sensor network address (addr) to a callback object provided by the UVPN endpoint. Later, the switch can resolve remote *virtual sensor nodes* using these associations between the addresses and the endpoints. This mechanism is functionally equivalent to the creation of a tunnel in a conventional VPN.

Therefore, to make the virtual network usable, it requires:

- At least one switch connected to the trunk network and at least to one sensor network, and
- At least an application or a service in the trunk network (e.g., on a desktop computer) that has the UVPN endpoint.

**Figure 4.**
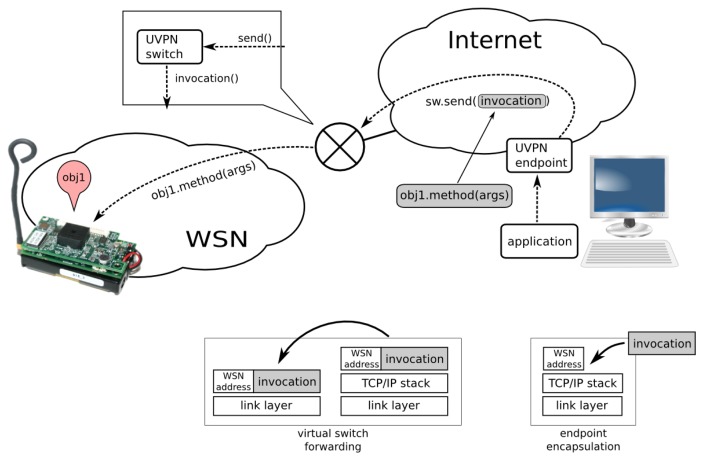
Ubiquitous virtual private network (UVPN) essential components and encapsulation.

A set of sensor nodes and the corresponding network interfaces are required, too. See Figure 4 for an example.

## 4. Use Cases

**Figure 5.**
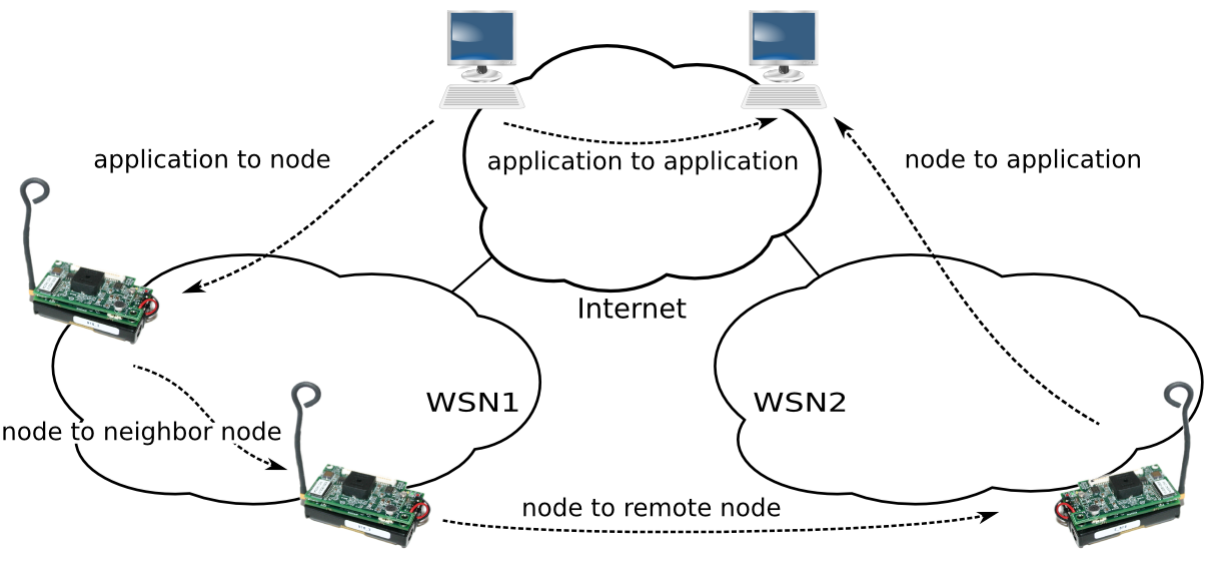
Possible invocation scenarios involving sensor nodes and applications in an heterogeneous inter-network.

This section discusses the different communication scenarios between node services and applications or between nodes themselves (see Figure 5).

### 4.1. Application to Node

In the simplest scenario, a client application running on a computer requests a remote sensor. In the application-level bridge-based approaches [15–17], the sensor network-specific protocols store the last measured sensor values in the bridge. Later, the client explicitly queries the bridge, giving some sensor identifier. This has been the cause of many important problems: bridge complexity implementation, lack of sensor node autonomy, single point of failure, *etc*.

With UVPN, the client (in the trunk network) performs a conventional remote object invocation on a proxy representing the remote sensor. The switch is also a distributed object, so the invocation for the sensor is encapsulated as a parameter of other object invocation (the switch). The UVPN endpoint knows (by configuration) where the *switch* (its addressing information) is and invokes the Transceiver.send() method, passing the entire client invocation message as an argument.

As a conventional distributed object, the switch receives the message and attempts to find (by means of the Switch.find() method) a *transceiver* (virtual node) for the node address specified as the first argument in the send() invocation.

In this case (the application to a node), the address is not found. The message is sent to the sensor network physical interface, directly connected to the computer running the switch service. The message is encapsulated with the sensor-network-specific stack and then goes to the air and should be received by the target node.

The sensor node is able to receive the invocation message from the frame and process a method invocation thanks to the picoObject approach. A middleware-level reply message may be generated if an answer is required. The reply message is encapsulated in a frame by the sensor node and sent to the air. The destination address of the reply is the *virtual node* running the client application. When the reply message arrives at the UVPN switch, it is forwarded using the same technique (very similar to the case in the next section).

Let us compare this mechanism with the way an Ethernet switch works. When the destination address of the frame is not known, the switch floods. Here, the registered *transceivers* might have the role of individual switch ports, and the wireless sensor network is similar to an “uplink switch”.

### 4.2. Node to Application

UVPN allows sensor nodes to behave as clients, that is, nodes can transparently invoke remote objects held in computers in the trunk network. This is a very rare feature in sensor network middlewares and communication frameworks. Usually, the sensor nodes may only send messages to other nodes or base-stations/bridges, but sending messages to devices outside the WSN usually requires *ad hoc* non-generic solutions.

In this case, the sensor node simply sends a conventional invocation preceded by the destination node address. The switch receives the message through the radio interface and searches for the destination address (with Switch.find()). In this scenario, the method returns a virtual node proxy. The switch uses it to forward the invocation to the computer.

The UVPN endpoint attached to the computer application receives the message and gives it to the object adapter. Finally, the corresponding servant method is executed.

### 4.3. Node to Neighbor Node

Of course, any node may send method invocations to any other neighbor in the same physical network using exactly the same mechanism described in the previous section. This means the invocation mechanism provides location transparency, *i.e.*, the client is not aware of the exact location of the destination object or of which type of node the target is (sensor node or computer application) or where it is (sensor or trunk network).

In this case, the switch will not find a remote virtual node, and it will send the message to the radio interface again. This is also useful when the sensor network does not implement multi-hop routing, because the switch will forward messages automatically. If destination nodes receive replicated messages through different paths, they are automatically discarded by just checking the sequence number in the header.

Messages sent to neighbor nodes pay the overhead of the middleware protocol message header (IceP). This is the price to obtain location transparency, but it is relatively cheap: approximately 25 bytes plus the object identity and the method name.

### 4.4. Node to Non-Neighbor Node

**Figure 6.**
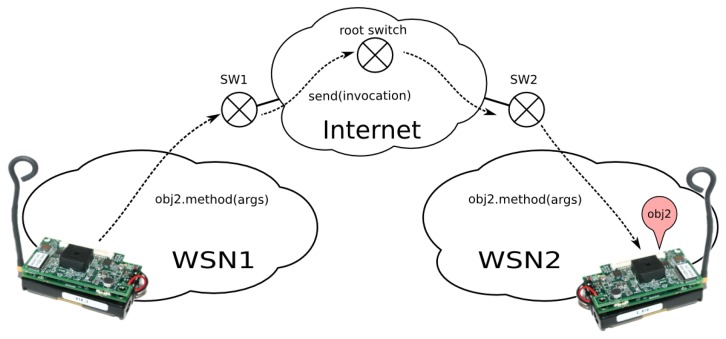
Invocation from a sensor node to a remote node using UVPN.

There is a more interesting case, which is very rare in previous works. Two or more distant sensor networks (with their respective UVPN switches) can be connected to the same trunk network (which may be the Internet). In this situation (see Figure 6), a sensor node may invoke other remote sensor node (in a different network) using switches to forward the message towards the trunk network. As explained before, this requires that the local switch knows whether the remote object is accessible through its own switch. This implies the registration of all sensor nodes in a central switch (the *root switch*). Obviously, we are talking about a hierarchical switching protocol. Local switches have a fallback switch (a *default path*) that knows where each sensor node is.

Figure 6 makes it clear that UVPN works as a tunneling protocol; so, in this case, just as in the previous one, a similar concept of conventional TCP/IP VPN protocols is used. The major difference is that the UVPN is based on an application-level protocol instead of a link or a network layer protocol.

The switch operation is a bit more complex when more than one sensor network participates in the communication. When a sensor node (such as a client) wants to send a message to another node, it builds the message as if the destination node were a neighbor, although it is in a different physical network. The local switch will receive the message, and it will check whether the destination address is registered on its table. If so, it sends the message using the associated *transceiver*. Otherwise, it will use the fallback switch to send the message (using the method, Transceiver.send()).

When this fallback switch (or the root switch, as stated above) receives a message, it will check if its destination is a registered one. If so, it sends the message again, using the matching transceiver. If the destination is unknown, then it will deliver the message to the rest of the configured transceivers, one on each gate (flooding). It only discards the arriving gate (to avoid loops).

When the last switch receives the message, it will check its table. If the destination is registered as a virtual node, it will send the message using the given transceiver. If not, the message will be sent to the other interfaces (*i.e.*, the radio interface, or to other registered switches, which are different from the arrival one).

### 4.5. Application to Application?

It is also possible to communicate no-sensor clients and objects using UVPN, but this does not provide any valuable difference with respect to using conventional middleware directly. Because the middleware supports several transports at the same time, it is more convenient to use TCP/IP endpoints. Without any loss of generality, this means that UVPN is used only when needed, *i.e.*, only when at least one sensor node is involved in the communication, either as a client or an object (server).

## 5. Simulation and Results

Several simulations have been built to demonstrate the correct operation of UVPN. They have been built in the OMNet++ (http://www.omnetpp.org/doc/omnetpp/manual/usman.html) discrete event simulator. An example smart-grid application was modeled to illustrate the operation of a network of sensor/actuator nodes (electrical sockets and bump lights) and an IP-based network, including conventional computers.

**Figure 7.**
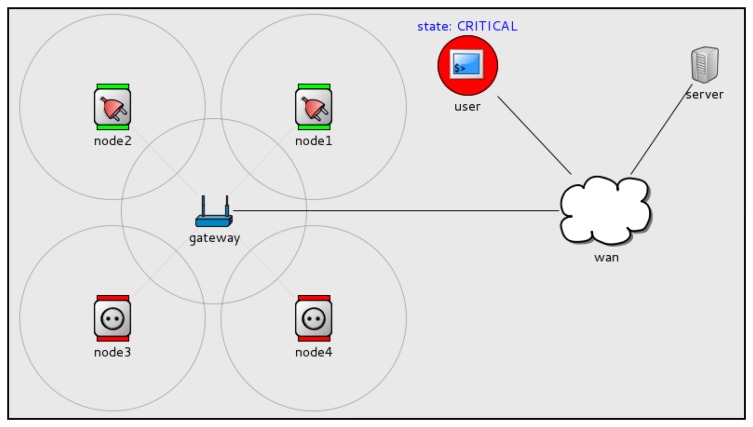
UVPN simulation with a smart-grid application example.

This smart-grid application (see Figure 7) monitors the total electrical consumption of a house to avoid current overload. Each socket measures the current through it and is able to interrupt the electrical line to turn the load connected to it on/off.

Three *load states* are defined:

- NORMAL: The total consumption is less than 70%.
- WARNING: The total consumption is between 70% and 90%.
- CRITICAL: The total consumption is greater than 90%.

Each time a user physically plugs/unplugs an appliance in a socket, the device sends a message to a monitor service (loadMonitor) running on a computer (server in the figure). The loadMonitor receives that message and decides the new state. It communicates load-state changes by sending messages to a specific event channel (using object-method invocations, too). It may include subscribers of any type (computer applications or sensor nodes), because all of them are perceived as distributed objects. Let us explain in detail the event sequence occurring in the simulation:

- In the initial state (NORMAL), there are four unplugged sockets.
- An appliance is plugged into the socket nodel. The socket measures the current and sends a message to the loadMonitor service.
- The loadMonitor service receives the message. It estimates that the system load exceeded the WARNING threshold, so it sets the load state to WARNING. An application (an event channel subscriber) running in a mobile device, tablet, *etc.*, may advise people (see the *user* shape) of the new state.
- Another appliance is plugged into socket node2. Again, the socket sends a notification. The loadMonitor detects that the load is above the CRITICAL threshold, so it sets the load state to CRITICAL. The sockets (that are channel subscribers) receive the message. Those that have nothing plugged into them immediately cut the line and turn on a red LED (Light-Emitting Diode) to visually advertise their state to the users. This can avoid the addition of a greater electrical load to the line, preventing overload.
- When some of the appliances are unplugged (or turned off), the loadMonitor can set the load state to lower states, allowing new appliances to be plugged or activated.

This example illustrates UVPN direct and duplex communication between sensor/actuator nodes and application objects running on conventional computers in the trunk network. The communication may be initiated by any party, and they all may act as clients or objects. Additionally, sensor nodes may act as publishers or subscribers of event channels.

**Figure 8.**
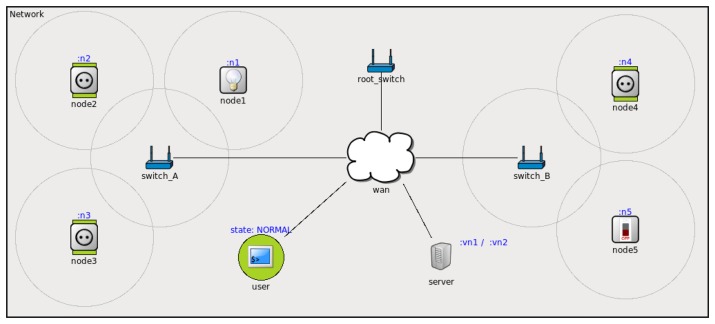
UVPN simulation: direct remote communication among sensor nodes in distant networks.

There is a more complete simulation involving two sensor networks connected to the same IP network through their corresponding UVPN switches (see Figure 8). This represents the same smart-grid application, introducing bulbs and electrical switches that are new electrical loads. To illustrate the communication among remote sensor/actuator nodes, the switch, *node5*, sends messages, *set()* true/false, to turn on/off the bulb, node1. As in the previous case, nodes 1–4 send load messages to the loadMonitor service and receive load state messages from the event channel. There are other nodes (e.g., user) that receive notifications from the loadMonitor service. In this case, the user will alert people about an overload.

The simulations source code, brief documentation and screencasts are available for download and watching at (http://arco.esi.uclm.es/uvpn).

The simulations attempt to provide an empirical validation of the proposed communication approach. The simulations use abstractions and message formats very close to those used in any object-oriented middleware, so it is relatively easy to build a functional implementation on an actual environment similar to the prototype described in Section 6. Although it is possible to take valuable measures about communication overhead or the number of messages, there are no equivalent approaches to compare under similar conditions.

## 6. Prototypes

**Figure 9.**
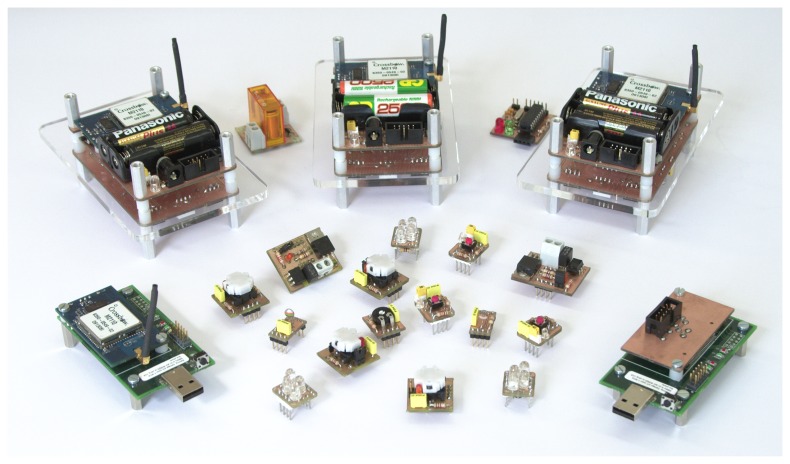
*Motebox* demonstration kit using UVPN.

UVPN has been implemented with the ZeroC Ice middleware in a demonstration kit called *Motebox* (see Figure 9). It is composed of a set of four Crossbow Iris motes, several hot-pluggable sensors and actuators, a programmer, a radio base station and a small PC for running common services and the switch software. The virtual node, which holds the clients and the servers, runs in a conventional laptop.

This kit is used to show various features upon access and interconnection between PC applications, middleware services and sensor nodes. More information and screencasts may be found at (http://arco.esi.uclm.es/motebox).

## 7. Conclusions and Future Work

It is interesting to analyze the differences in relation to [2]. It encapsulates the entire sensor network protocol stack, which implies the requirement of providing specific support for each sensor network protocol stack at *virtual nodes*. With UVPN, it does not matter which sensor network protocol stack is used, as long as all peers use the same inter-ORB protocol (at the application layer) and the same addressing scheme; that is, the typical requirements of an inter-network protocol. Thus, UVPN is, in many senses, an object network protocol.

Furthermore, using the inter-ORB protocol (by means of an object-oriented middleware), provides a high-level, well-known, well-documented programming paradigm and an application-specific API (Application Programming Interface), but keeps the gateways generic and straightforward. The middleware also provides valuable common services, such as object persistence, indirect binding, location transparency, server deployment and many other advanced features.

As far as we know, UVPN is the first solution able to facilitate the communication between sensor or actuator nodes with software objects running on conventional computers in a seamless and decoupled way, without the application-specific delegates, application bridges or *ad hoc* protocols, despite the heterogeneous networks and protocols that interconnect sensor network fragments (including the Internet). However, there is a constraint: all sensor nodes must use the same physical addressing scheme. As ongoing work, we are interested in solving that limitation by generalizing the UVPN approach using a global addressing scheme (see Section 3) and providing homogeneous dynamic routing mechanisms through massively heterogeneous networks.
